# Taking action towards climate-resilient, low-carbon, health systems: Perspectives from Canadian health leaders and healthcare professionals

**DOI:** 10.1177/08404704241252032

**Published:** 2024-05-13

**Authors:** Brittany Barber, Daniel G. Rainham, Peter Tyedmers, Trevor Vandertuin, Gillian Ritcey, Sean D. Christie

**Affiliations:** 1 3688 Dalhousie University, Halifax, Nova Scotia, Canada.

## Abstract

Climate change poses significant public health and health system challenges including increased demand for health services due to chronic and acute health impacts from vector-borne diseases, heat-related illness, and injury from severe weather. As climate change worsens, so do its effects on health systems such as increasing severity of weather extremes causing damage to healthcare infrastructure and interference with supply chains. Ironically, health sectors globally are significant contributors to climate change, generating an estimated 5% of global emissions. Achieving “net zero” health systems require large-scale change with shared decision-making to coordinate a pan-Canadian approach to creating climate-resilient and low-carbon healthcare. In this article, we discuss healthcare professionals’ and health leaders’ perceptions of responsibility for practicing and advocating for climate-resilient and low-carbon healthcare in Canada.

## Introduction

Despite calls to action to address the accelerating climate crisis,^1^ there are few coordinated efforts in Canada that raise awareness of the scale of action required to reduce health impacts from climate change and mitigate Greenhouse Gas (GHG) emissions from the delivery of health services.^2-4^ Lack of urgency to adapt resource-intensive health systems is a major concern.^5,6^ Canada, Australia, the United States, and Switzerland are the top four worst health sector emitters per capita, emitting between 30 and 50 times more per capita than does India.^7^ Achieving “net zero” health systems—that is, reducing health sector GHG emissions as much as possible, and offsetting any remaining emissions^8^—will require shared responsibility across levels of government and health authorities to align decision-making and implement effective low-carbon healthcare practices.^9^ This presents challenges for Canada’s decentralized healthcare systems where provincial-territorial regions are responsible for one of the largest service sectors, employing more than 2 million people and accounting for one-third of all provincial-territorial expenditures.^10^ Cultivating shared responsibility will be a key lever for progress in the development of a pan-Canadian approach to creating climate-resilient and low-carbon health systems.

Impetus for addressing GHG emissions from healthcare has shifted between health professionals^5^ and health leaders,^4^ making it challenging to take coordinated action.^11,12^ Efforts to mitigate carbon emissions by Canadian health organizations^13^ demonstrate the leadership required to facilitate health system environmental stewardship. However, multiple competing health system priorities and limited healthcare resources makes it challenging for health organizations to assess the magnitude of climate change impacts and coordinate high-impact solutions.^11^ Given recent prioritization of healthcare professionals’ awareness of climate change and impacts on health systems,^14–16^ we aimed to better understand perceptions of responsibility for practicing and advocating for climate-resilient and low-carbon health systems among healthcare professionals and health leaders.

## Methods

We conducted surveys of Canadian healthcare professionals, including clinicians from surgery (any speciality) and anaesthesia (n = 100), undergraduate medical students (n = 31), and interviews with ten health leaders in Nova Scotia. Survey and interview questions focused on understanding the importance of environmentally sustainable health systems, as well as responsibilities and challenges associated with adapting health systems to improve climate-resilient and provision of low-carbon healthcare services.

## How important is climate-resilient, low-carbon health systems to health leaders and healthcare professionals?

Healthcare provision, encompassing public/private delivery of care, services, and supplies, is a significant source of greenhouse gas emissions.^2^ Estimated at 5% of Canada’s total emissions, health systems rival those of large economic sectors including marine and aviation.^17^ Ironically, the GHGs emitted from the delivery of healthcare will lead to population health impacts that will be borne differently across populations, and additional demands on the healthcare system. Future generations are at highest risk for climate-related health conditions such as onset and exacerbation of chronic conditions including cardiovascular and respiratory diseases due to worsening air quality, human-made air pollution, airborne allergens, and wildfire smoke.^18^ An estimated 4 million new paediatric asthma cases globally are attributable to traffic-related air pollution annually, the majority occurring in urban centres.^19^ Further, increasing frequency and severity of weather extremes from climate change can damage healthcare infrastructure, interfere with supply chains, and increase demand for healthcare services (e.g., heat stress, vector-borne diseases, and respiratory diseases from reduced air quality). In the past decade, extreme weather events across provincial-territorial regions in Canada caused disruptions to delivery of healthcare and damage to healthcare infrastructure including acute-care facilities, electricity and water services, and roadways. For example, in 2021, widespread flooding in southwestern British Columbia caused devastating damage to healthcare infrastructure including mudslides wiping out roadways and restricting travel in and out of communities to access healthcare, loss of electricity and water damage requiring hospital closures and evacuations,^20^ and disruptions to healthcare supply chains including medication shortages and inability to transport medications and receive treatments.^21^ Health implications of extreme weather events are disproportionately felt by vulnerable and disadvantaged populations^22^ and commonly lead to higher prevalence of chronic depression, anxiety, and reduced quality-of-life.^20^ It is estimated that GHG emissions and pollution from Canada’s healthcare sector result in an annual loss of 23,000 (4,500-610,000) disability-adjusted life years.^17^

Health leaders we interviewed described addressing climate health and environmental impacts of health systems as “extraordinarily important” (P01). Awareness of environmental impacts of health systems was described through direct and indirect sources such as, “healthcare facilities and the industry itself are one of the most substantial producers of carbon emissions, just based on what their power consumption is, heat generation, fuel source, plus the use of disposable items” (P02). All but seven healthcare professionals (95%) surveyed agreed climate change is an important societal issue. However, when asked to rank professional practice and health system goals relating to the care of patients, all healthcare professionals reported environmental impact of care as *least* important and medical surgical outcomes and safety of patients as most important. This is unsurprising given the mounting pressures on health systems and healthcare professionals, including chronic workforce shortages, patients with complex and high acuity needs, long wait times and limited access to care, and lack of investment in preventive health.^23^ Although health leaders and healthcare professionals reported a good understanding of climate health and its consequences, shared responsibility for integrating climate health mitigation with other health system objectives is lacking.

## Who shares responsibility for advancing climate-resilient and low-carbon health systems?

Limited awareness of the health-related co-benefits from action for climate health mitigation and adaptation makes it challenging to coordinate action. Top-down governance of regulatory policies and bottom-up advocacy of practice changes have been adopted by countries globally to achieve GHG emission reductions.^9^ Strong governance structures and policies are important for coordinating shared responsibility and reducing GHG emissions from healthcare sectors, as demonstrated by the United Kingdom’s centralized approach to achieving a net zero National Health Service (NHS).^24^ The NHS’s governance strategy for achieving net zero health systems includes launching a Greener NHS program,^24^ Health and Care Act legislation,^25^ and mobilizing workforce action to decarbonize facilities (i.e., construction of net zero hospitals and heating and lighting) and healthcare (i.e., low-carbon medicines and supply chain, zero-emissions transport and travel, and digital care pathway redesign).^24^ Further, Germany’s bottom-up approach to net zero health systems includes grassroots action from sustainability champions at individual hospitals and organizations.^9^ Germany’s KLIK Green organization provides training to clinical professionals and building capacity with teams of interprofessional climate managers on behalf of their institutions.^26^ Canada’s provincial-territorial health system governance makes it challenging to employ a coordinated approach to regulating GHG emissions, such as enforcing national standards for decarbonizing health systems. As such, Canada will likely follow a similar decentered approach, as in Germany, to achieve net zero health systems^9^ where all health centres have institutional autonomy with no overarching obligation or strategy for operating climate-resilient and low-carbon healthcare facilities.

The lack of provincial-territorial strategies supporting low-carbon decision-making signifies that responsibility for coordinating environmentally sustainable health systems falls to local health leaders, healthcare professionals, and the private sector. Such strategies “would hold us accountable so we couldn’t spend it on something else, because the trouble with health is, if you give us more money it will be spent, right? I think some targeted investment that would be the area that government could certainly play a role” (P04). Healthcare systems can play a pivotal role in promoting actions towards reductions in GHG emissions. “We should be leaders, we should be mentors, and we should be modelling it for the rest of our communities” (P08). Investments in healthcare should also prioritize low-carbon, climate-resilient infrastructure. “The standard that we have to follow is LEED silver of design for environmental impact. However, we should be going for gold and platinum, beyond silver. But that is not dictated federally or provincially, and the government is always in business of cost containment so they’re going to go to the lowest standard that’s more economical” (P06).

Coordinated action from healthcare sectors is another key lever for mitigating environmental impacts. Of the healthcare professionals we surveyed, more than 75% believed that the healthcare sector has a responsibility to practice and advocate for low-carbon healthcare. An even greater proportion of healthcare professionals (84%) reported interest in personally championing low-carbon healthcare practices in the workplace or, for medical students, in their future practice. Despite this high interest, coordinated action for implementing low-carbon healthcare practices is required. Knowing what low-carbon practice change options are available can be challenging. For example, most healthcare professionals reported they were unaware of environmentally sustainable measures in place to dispose of anaesthetic gases (77% don’t know) and efforts to minimize waste in operating rooms (72% don’t know, no efforts). Solutions may include integrating carbon emissions data into health technology assessments, adapting procurement practices to give preference to products with lower environmental impact, or adopting practice changes that reduce waste and avoid unnecessary treatment.

Advancing a culture of climate-resilient and low-carbon healthcare systems requires investment in training of future healthcare professionals. A survey of 84 international health professional institutions reported 63% offer climate health education as part of a required core course, with an additional 11% of institutions under discussion of adding climate health course offerings.^27^ Although efforts are ongoing with the Canadian Federation of Medical Students,^28^ Canada is falling behind in implementing climate and planetary health curriculum changes across health professional training programs.^29^ Of medical students we surveyed, 84% reported climate health was a low priority within the medical school curriculum. Climate and planetary health education and training are an important part of a multi-pronged strategy for increasing awareness and shared responsibility for taking action to improve climate-resilient and low-carbon healthcare practice.

## What challenges persist to achieving climate-resilient and low-carbon health systems in Canada?

High performing, low-carbon health systems aim to enhance high-quality care optimizing patient safety and health outcomes, adapt to population needs from climate change-related diseases and injuries, and reduce costs by investing in climate-resilient healthcare facilities. Many healthcare systems are challenged with adapting resource-intensive health facilities that require high energy intensity and produce significant waste from single-use medical supplies, an estimated 13.2 kg of waste per patient per day.^30^ Challenges to decarbonizing healthcare systems are unique to each healthcare facility, such as challenges to integrating climate policies into governance and leadership, opting for renewable energy sources, supply chain rigidity, public awareness, and financial resources. Key barriers and enablers to implementing low-carbon healthcare practices reveal most barriers exist at the institutional level including lack of vision and leadership, organizational readiness, lack of defined targets, costs, supplier standards, conflicting protocols, and inadequate staffing.^31^

Health leaders discussed several challenges to achieving climate-resilient and low-carbon health systems in Nova Scotia due to: ageing infrastructure; inefficiencies in care delivery; upfront investments and short-term cyclical funding resources; competing provincial priorities such as access to care and chronic workforce shortages; greening the grid with renewable energy; advocating for upstream investments within a crisis-oriented health system; and systems of accountability from national and provincial standards with incentives or penalties. Healthcare systems are significantly challenged by, “…aging infrastructure, you know the biggest challenge associated with all of it is spending money on infrastructure” (P10). Furthermore, “the infrastructure side of things requires time and disruption, you can’t close the hospital while you retrofit to be more environmentally sustainable” (P09), and “where is the energy coming from? If it’s solar, great. But if it’s still relying on coal-fired power, there are some big external factors” (P05). Another key challenge, “it’s money, money, money right? It’s always the budget is the bottom line, and although doing things differently is often cost-savings, it takes time to do things differently. But the way governments run they generally will never have more than a 4-year strategy. So, it’s really hard to get that vision and programming in place when your funder will never have more than a 2-year vision” (P03).

Healthcare professionals reported challenges to decarbonizing healthcare including lack of prioritization across interprofessional teams, lack of leadership commitment, juxtaposition of infection prevention and control practices for patient safety, cost, lack of low-carbon supply chain and procurement options, and the creation of low-carbon practice changes that are easier and efficient.

## More than just money: What is the long-term viability of health systems?

The long-term viability of healthcare systems is uncertain, in part due to unsustainable costs, increasing demand for healthcare resources, and the integration of expensive technology into clinical practice. Annual global healthcare expenditures have steadily increased, doubling between 2000 and 2018 from $4.1 trillion (approximately 8.3% of global GDP) to $8.3 trillion (approximately 10% of global GDP).^32^ Furthermore, greater healthcare spending over this time period is correlated with environmental impacts, including an estimated 29% increase in global GHG emissions.^33^ Control of healthcare spending will require strategic investments towards disease prevention and high achieving health systems, optimizing the triple bottom line for improving quality of patient care and health outcomes, reducing healthcare costs, and mitigating environmental harms from care delivery.^34^ Common misconceptions that climate-resilient and low-carbon measures are cost prohibitive may play a role in preventing healthcare organizations from taking action and planning for net zero practice change.^35^ But it is more than just about money.

There are multiple social, economic, and ecological co-benefits from high performing, low-carbon, climate-resilient health systems. Almost two-thirds of healthcare professionals we surveyed perceived that prioritizing environmentally sustainable healthcare would have a *neutral effect* on the quality of patient care. Cataract surgery is one of the most common surgeries performed globally, contributing an estimated 180 kg of CO_2_-equivalent GHG per patient eye in the United Kingdom.^36^ Using an assembly line model of cataract surgery, Aravind Hospital in India has pioneered a low-carbon approach at no additional costs, producing a mere 6 kg of CO_2_-equivalent GHG per patient eye.^35^ Aravind optimized the physical layout of operating rooms, efficiency of task shifting, reduced waste, switched to reusable medical equipment and surgical gowns, and maintained lower intraoperative complication rates.^36^ Further, implementation of Immediate Bilateral Cataract Surgery (ISBCS)—phacoemulsification with intraocular lens placement in both eyes of the patient on the same visit in the same operative setting—at health centres in Spain,^37^ Finland,^38,39^ and Sweden^40^ show faster patient rehabilitation, improved cost effectiveness, elimination of duplicate operative services reducing GHG emissions from patient travel, and building and energy use.^41^ Interestingly, health leaders highlight additional co-benefits “by virtue of some of the environmentally sustainable changes that could be made, it’s more of a morale booster as opposed to a morale reducer” (P10). Demonstrating the triple bottom line of healthcare innovations that do more with less for more people is important for advancing the development and uptake of low-carbon healthcare practices.

## Conclusion

Over the last four decades, there have been several calls for stronger leadership and coordinated action to drive progress towards climate change commitments in Canada.^3^ Current efforts towards a transformation to high performing, low-carbon healthcare systems remain largely disjointed across provincial-territorial health authorities and agencies. While most healthcare professionals and system leaders agree that climate health is extraordinarily important, shared responsibility for reducing environmental impacts with other health system objectives is lacking. Surveys with one hundred Canadian healthcare professionals and interviews with ten Nova Scotia health leaders provide an important starting point for understanding the responsibilities and challenges associated with adapting health systems to improve provision of low-carbon healthcare. However, this small sample is not representative of the Canadian healthcare workforce and generalizability of results to the broader context of diverse provincial-territorial health systems may be limited. Strategic efforts are underway in Nova Scotia, Ontario and British Columbia to identify environmental impacts from care and to support Canada’s healthcare community towards high-quality, low-impact, climate-resilient care. These efforts should be expanded, networked and adequately supported to lower Canada’s global standing among countries with high-emitting healthcare systems. Efforts to strengthen the capacity of interprofessional healthcare communities with adapting climate-resilient and low-carbon health systems are demonstrated by pan-Canadian networks such as the Collaborative Centre for Climate, Health and Sustainable Care (CASCADES) at the University of Toronto,^42^ Planetary Healthcare Lab at the University of British Columbia,^43^ Healthy Populations Institute Creating Sustainable Health Systems in a Climate Crisis at Dalhousie University,^44^ and the Canadian Coalition for Green Health Care.^45^ Advocating for greater accountability between federal-provincial policies and regulations has strong potential for advancing progress, such as embedding targets for reducing GHG emissions within Canada’s accreditation standards for healthcare facilities. Backing climate targets with strong plans and effective implementation is essential not only for increasing shared responsibility but also for ensuring the long-term viability of the healthcare sector, the Canadian population, and the planet.
